# Gut microbiome differences between wild and captive black rhinoceros – implications for rhino health

**DOI:** 10.1038/s41598-019-43875-3

**Published:** 2019-05-28

**Authors:** Keylie M. Gibson, Bryan N. Nguyen, Laura M. Neumann, Michele Miller, Peter Buss, Savel Daniels, Michelle J. Ahn, Keith A. Crandall, Budhan Pukazhenthi

**Affiliations:** 1 Computational Biology Institute, The Milken Institute School of Public Health, The George Washington University, Washington, DC USA; 2 Department of Biological Sciences, The George Washington University, Washington, DC USA; 3 Department of Environmental and Occupational Health, The Milken Institute School of Public Health, The George Washington University, Washington, DC USA; 4 DST-NRF Centre of Excellence for Biomedical Tuberculosis Research; South African Medical Research Council Centre for Tuberculosis Research; Division of Molecular Biology and Human Genetics, Faculty of Medicine and Health Sciences, Stellenbosch University, Cape Town, South Africa; 5 South African National Parks, Veterinary Wildlife Services, Kruger National Park, Skukuza, South Africa; 6 Department of Botany and Zoology, University of Stellenbosch, Private Bag X1, 7602 Matieland, South Africa; 7 Department of Epidemiology and Biostatistics, The Milken Institute School of Public Health, The George Washington University, Washington, DC USA; 8 Smithsonian’s National Zoo and Conservation Biology Institute, Front Royal, VA USA

**Keywords:** Microbiota, Biodiversity

## Abstract

A number of recent studies have shown the importance of the mammalian gut microbiome in host health. In the context of endangered species, a few studies have examined the relationship between the gut microbiome in wild versus captive populations due to digestive and other health issues. Unfortunately, the results seem to vary across taxa in terms of captive animals having higher, lower, or equivalent microbiome diversity relative to their wild counterparts. Here, we focus on the black rhinoceros as captive animals suffer from a number of potentially dietary related health effects. We compared gut microbiomes of wild and captive black rhinos to test for differences in taxonomic diversity (alpha and beta) and in functional diversity of the microbiome. We incorporated a more powerful metagenomic shotgun sequencing approach rather than a targeted amplification of the 16S gene for taxonomic assignment of the microbiome. Our results showed no significant differences in the alpha diversity levels between wild and captive black rhinos, but significant differences in beta diversity. We found that bacterial taxa traditionally associated with ruminant guts of domesticated animals had higher relative abundances in captive rhinos. Our metagenomic sequencing results suggest that unknown gut microbes of wild rhinos are being replaced by those found in conventional human-domesticated livestock. Wild rhinos have significantly different functional bacterial communities compared to their captive counterparts. Functional profiling results showed greater abundance of glycolysis and amino acid synthesis pathways in captive rhino microbiomes, representing an animal receiving sub-optimal nutrition with a readily available source of glucose but possibly an imbalance of necessary macro and micronutrients. Given the differences observed between wild and captive rhino gut microbiomes, we make a number of recommendations for potentially modifying captive gut microbiome to better reflect their wild counterparts and thereby hopefully improve overall rhino health in captivity.

## Introduction

From more than 100,000 free-ranging African black rhinos in the 1960s, this critically endangered species has declined by more than 90% to approximately 5,000 animals today^1^. On average over 1,000 rhinos are poached annually in range countries that include South Africa, Namibia, Kenya, and Zimbabwe^1^. Currently, fewer than 100 black rhinos (southern and eastern sub-species combined) reside in zoological institutions in North America as a reservoir against potential extinction^2^. However, the *ex situ* population experiences its own threats to survival, including a myriad of unusual disease syndromes not generally described in the wild^3–10^, as well as poor reproduction^11,12^ and fragmented populations^3^. Across mammals, recent studies have suggested that microbiome differences between wild and captive populations may influence overall health in general and digestive and immune functions in particular^4^.

A number of recent studies have identified differences between wild-captive populations or wild-domesticated populations of mammals. For example, Schmidt *et al*.^10^ examined microbiome diversity between wild and captive individuals of deer mice (*Peromyscus maniculatus*) and found that mice from natural environments had more diverse gut microbiome communities, but that gut microbiomes were more similar by like environments rather than wild versus captive. Likewise, Clayton *et al*.^5^ showed that in nonhuman primates, captivity ‘humanizes’ their microbiome showing a convergence to gut microbiome reflective of the human gut via replacement of diverse microbial diversity across species. Wasimuddin *et al*.^13^ compared wild versus captive cheetahs and reported differences in gut microbiome between kin and nonkin individuals as well as a higher incidence of pathogenic strains in captive cheetahs. McKenzie *et al*.^4^ took a broader taxonomic approach and examined gut microbiome diversity across wild versus captive populations of a variety of mammals. They investigated trends across six mammalian orders and found alpha diversity between wild and captive populations consistent across some mammalian hosts, decreased in captive populations in some hosts, and increased in one host – namely, the rhinoceros^4^. Interestingly, this conclusion was a combined analysis across both white and black rhinos with limited sampling (especially unbalanced sampling in the black rhino with six captive but only one wild individual). Clearly, the jury is still out on the impacts of captivity on gut microbiome diversity, and it may very well be that the impact is species specific.

These previous studies testing the associations of gut microbiome diversity in wild versus captive populations have focused on targeted amplicon sequencing of a single gene, 16S rRNA, to characterize the gut microbiome as a metataxonomic approach. While less cost effective, taking a shotgun metagenomic approach to characterizing the gut microbiome provides a number of advantages^6^. First, the metagenomic approach does not rely on PCR and is therefore not subject to PCR artifacts^7^. Metagenomics provides greater resolution (down to strain level) compared to metataxonomic approaches. Metagenomics can also identify virus, fungus, and other taxa in addition to bacteria – all in the same sequencing run^8^. It also provides for greater functional assignments as the data survey across the genome, not a single ribosomal gene^9^. Given these advantages of metagenomics and the lack of consensus on the impact of captivity on gut microbiome coupled with our focus on black rhino health in captivity for conservation options, we applied metagenomic sequencing to wild and captive black rhino fecal material to characterize microbiome diversity as well as test for differences between wild and captive animals from both taxonomic and functional perspectives. We then make recommendations based on this collective information for adjusting diet to create a normative gut microbiome, which in turn may promote better black rhino health.

## Results

### Gut microbiome

#### Read mapping and extraction approaches

All samples were sequenced to a depth of at least 6.9 million paired-end reads per sample, with an average of 12,479,613 paired-end reads. Very few reads were discarded during quality trimming (3.16%); the post QC average was 12,085,574 paired-end reads per sample.

Overall, low mapping rates (<10%; number of mapped reads/number of cleaned reads) were observed across all samples and across all three metagenomic mapping software platforms (PathoScope, Kraken, and Centrifuge; Table 1). Although PathoScope mapped fewer reads than Kraken and Centrifuge, PathoScope provided better resolution at the lower taxonomic levels. Prokaryotes made up most of the known mapped reads (average 3% of reads per sample with PathoScope). Rhino and human contamination in the reads were low (~1% average PathoScope mapped reads, respectively). Despite the wild rhino fecal samples having plant material visibly present in the DNA/RNA Shield, very few reads mapped to plant genomes from the Gramene database (average 0.02% or 2,800 reads). On average, 97% of all reads were unmapped, a surprisingly high proportion of reads that suggests our reference databases are not robust to perhaps novel taxa coming from black rhino guts. A slightly higher proportion of unmapped reads were in the wild rhinos compared to captive (average 12,090,472 vs 11,625,025 reads, respectively); one of the wild rhinos (R08) had 36.3% of its reads map to the rhino reference genome, accounting for over 3.5 million reads mapped. This was the most reads mapped for any rhino to any database. Wild rhinos had over a 2,000-fold increase over captive rhinos in reads that mapped to the rhino reference genome (average = 408,545 reads vs 156 reads, respectively). Notably, the captive samples (R21-R28) had consistently higher proportions of assigned reads for each sample (average 460,939 reads) compared to wild samples (R01-R20; average 287,611 reads).

**Table 1:** Average mapping percentage for all metagenomic mapping software platforms.

Database	Wild Rhinos			Captive Rhinos		
	PathoScope	Centrifuge	Kraken	PathoScope	Centrifuge	Kraken
Rhino	0.52%	NA	NA	0.00%	NA	NA
Human	0.00%	NA	NA	0.00%	NA	NA
Prokaryotes	2.06%	8.34%	2.97%	3.29%	10.06%	3.55%
Eukaryotes	NA	4.49%	0.13%	NA	3.96%	0.17%
Other	0.10%	0.05%	0.00%	0.13%	0.05%	0.00%
Unknown	97.21%	87.17%	96.91%	96.55%	85.93%	96.28%

The operational taxonomic unit (OTU) richness was inconsistent across samples and was influenced by extraction kit used. The ZymoBIOMICS-extracted samples had more OTU hits on average compared to the MoBio PowerFecal-extracted samples, regardless of origin (wild vs captive; p = 0.099). PathoScope assigned a larger proportion of reads to the set of wild samples (R2-R11) that were stored in Zymo DNA/RNA shield and extracted using the ZymoBIOMICS DNA Miniprep kit (average 447,354 reads) than the wild samples frozen and extracted with MoBio PowerFecal kit (average 155,729 reads). The Zymo wild samples had an average of 350 OTUs at the species level, whereas the MoBio wild samples had 250 OTUs (p = 0.036). Thus, collection method did influence the microbiome composition. Observed communities differed markedly between the MoBio-extracted and Zymo-extracted wild samples (PERMANOVA, p < 0.0001). However, several bacterial species such as *Bacteroides fragilis* and *Escherichia coli* were present in both MoBio- (R01-R08) and Zymo-extracted (R11-R20) samples.

#### Taxonomic composition and diversity

**Figure 1:**
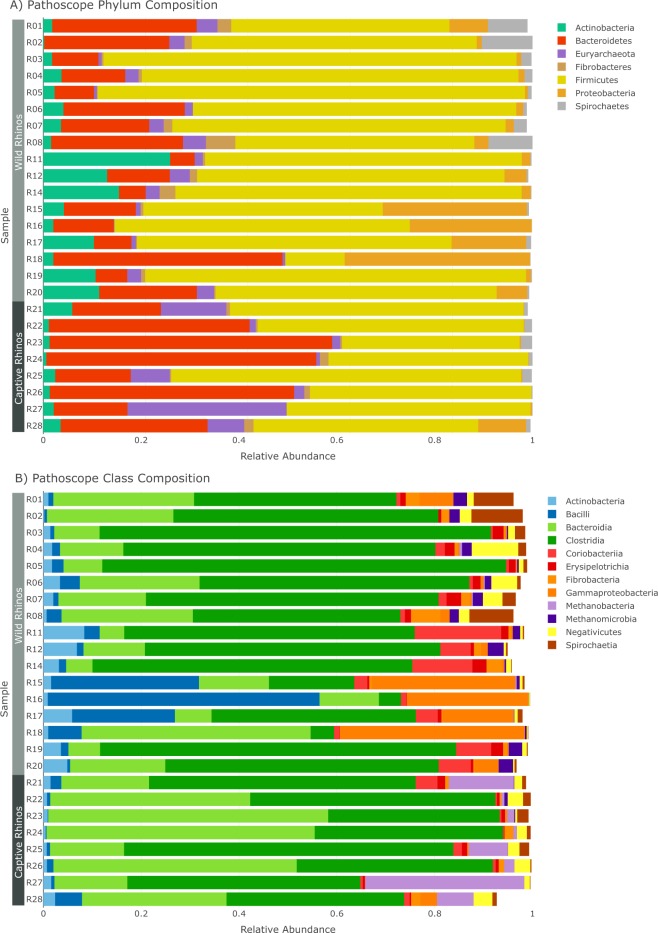
Rhino microbiome composition, as determined by PathoScope, broken down by (**A**) phylum and (**B**) class, grouped by wild versus captive host. Empty space represents bacterial reads not identified at the corresponding taxonomic rank. Taxa representing less than 1% of reads on average and less than 5% across all samples were filtered out for the sake of visualization.

Differences in higher level taxonomic community composition were observed between wild and captive rhinos (Fig. 1A,B). Specifically, Proteobacteria was more abundant in wild compared with captive rhinos while Bacteroidetes was abundant in captive rhinos. The wild rhinos showed a higher incidence of phylum Actinobacteria and class Acidaminococcales, while their captive counterparts had elevated levels of Euryarchaeota. Additionally, the class Erysipelotrichia was present in five out of the eight captive rhinos, but in only a single wild rhino.

**Figure 2:**
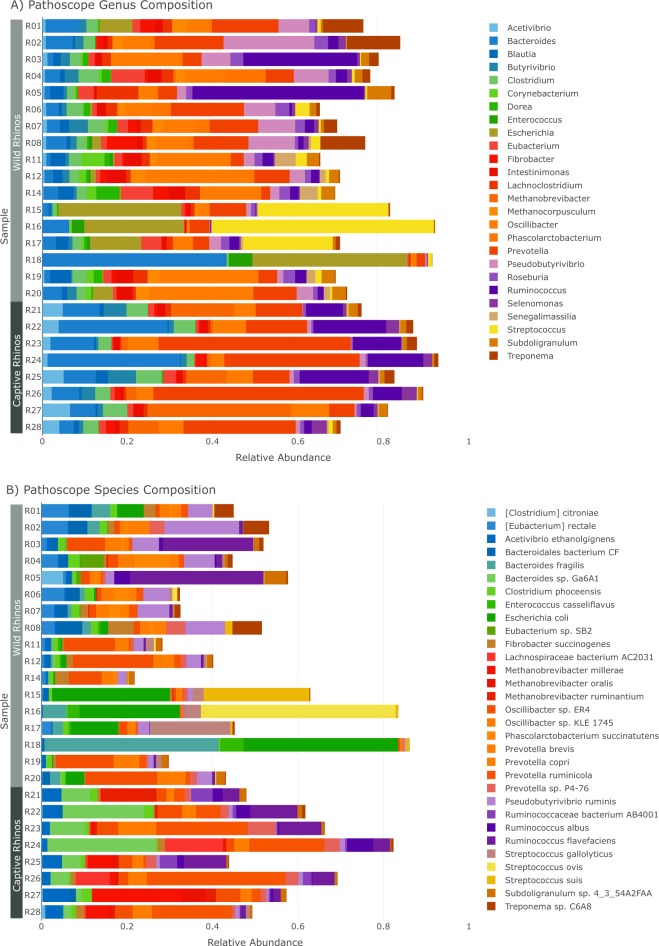
Black rhino bacterial microbiome composition, as determined by PathoScope, broken down by (**A**) genus and (**B**) species, grouped by wild versus captive host. Empty space presents bacterial reads not identified at the corresponding taxonomic rank. Taxa representing less than 1% of reads on average and less than 5% across all samples were filtered out for the sake of visualization.

At the phylum and class level, abundances of a few bacteria distinguish the rhino microbiomes based on wild versus captive status. Analysis at the genus level revealed greater differences between the wild and captive rhinos (Fig. 2A). Genera *Escherichia*, *Oscillibacter*, *Pseudobutyrivibrio*, and *Treponema* were higher in the wild rhinos, while *Bacteroides* and *Prevotella* were increased in all the captive counterparts. However, both wild and captive rhinos expressed the major groups of microbes for digestion (cellulolytic, amylolytic) but were represented by different species (i.e., functionally similar, but taxonomically distinct microbiomes; Fig. 2B). There also were a number of species that were differentially abundant between wild and captive rhinos. For example, the methane producing bacteria *Methanocorpusculum bavaricum* (p = 0.0059) was more abundant in wild rhinos, whereas captive rhinos contained *Methanobrevibacter ruminantium* (p = 2.06e-28). *Bacteroides fragilis* (p = 0.0005), *Steptococcus suis* (p = 3.94e-15), and *Escherichia coli* (p < 0.001; strains range from p = 4.8e-12 through 3.5e-05) also were found to be high in wild rhinos, whereas *Ruminoccus albus* (p = 1.73e-27) and *Prevotella ruminicola* (p = 0.0003) were highly abundant in animals maintained in captivity. However, a quarter of the assigned OTUs were not able to be assigned down to species level, again suggesting the inadequacy of the reference database for microbes.

Common taxa were found across the wild and captive rhinos, respectively, suggesting a core rhino microbiome based on captivity status (Supplementary Table S1a,b). The phylum Firmicutes dominated the microbiome of both the wild and captive rhinos, which comprised 32.7% and 20.8% of total mapped reads and 51.1% and 48.0% of the core microbiome, respectively. However, the next leading dominate phyla in the wild rhinos were Proteobacteria (23.6%) and Bacteroidetes (17.6%), while Bacteroidetes was the second dominant phylum in the captive rhino microbiome (42.4%).

**Figure 3:**
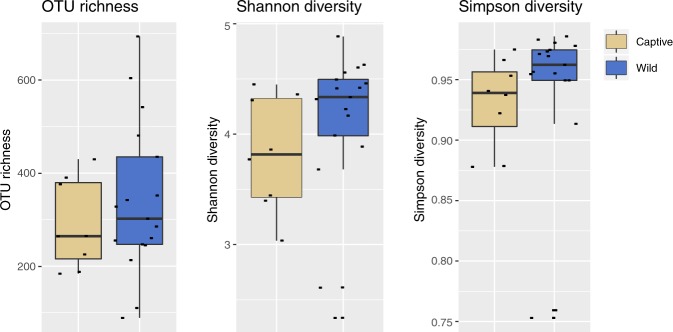
PathoScope sample-level OTU richness and diversity (Shannon and Simpson indices) of the wild and captive rhino populations.

**Figure 4:**
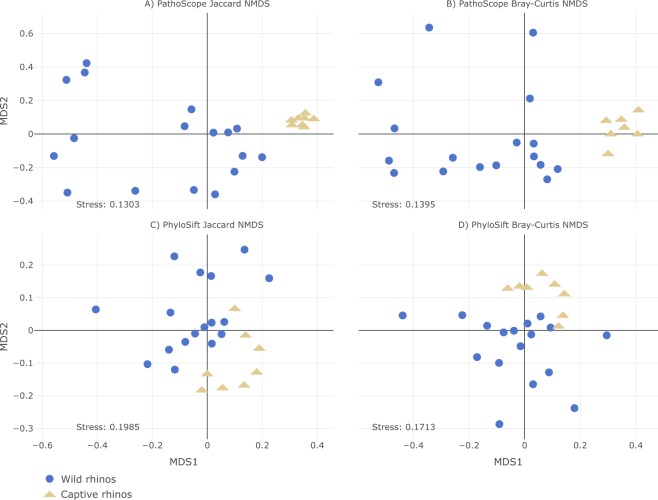
Non-metric multidimensional scaling plots of PathoScope data using Jaccard distances (**A**) and Bray-Curtis distances (**B**) and of PhyloSift data using Jaccard distances (**C**) and Bray-Curtis distances (**D**).

Although the taxonomic composition of the rhinos shows differences between wild and captive gut microbiomes, alpha diversity measures between the two groups were similar. However, the observed species richness (p = 0.082) did indicate that the wild rhinos have a higher median observed OTU richness (~335) compared to the captives (~220) (Fig. 3), which is consistent with all the taxonomic results. Both the Shannon (p = 0.36) and Simpson (p = 0.69) diversity indices indicate that the rhinos show high diversity, independent of their origin (wild vs captive), with wild rhinos showing slightly (but not significantly) higher diversity (Fig. 3). For all three alpha diversity measures, the samples derived from wild rhinos displayed greater variance along with more outliers compared to the captive samples and clustered together based on their origin (wild vs captive; Fig. 4). These patterns were consistent across both the Jaccard and Bray-Curtis indices. Furthermore, these patterns were consistent between the PathoScope (Fig. 4A,B) and PhyloSift results (Fig. 4C,D). The dissimilarity metrics, measured separately with Bray-Curtis, Jaccard, and Jensen-Shannon (JSD) indices with 10,000 permutations, were all highly significant (PERMANOVA, p = 9.999e-05), representative of the centroids being different between the two groups. This is indicative of the two groups having distinct and different communities. Together the ordination plots and PERMANOVA indicated that the microbiomes of rhinos were more similar to other rhinos with the same captivity status, with wild rhinos displaying considerably more variation and range than captive rhinos.

#### Functional analysis

A total of 39 gene ontology (GO) terms were found to be differentially abundant between wild and captive rhino microbiomes with a Q-value less than 0.05 (Supplementary Table S2). The majority of the GO terms were positiviely associated with captive rhinos’ microbiomes. A total of 127 pathways were differentially abundant, however only two pathways (PWY_5103 L_isoleucine_biosynthesis_III and PWY_6121 5_aminoimidazole_ribonucleotide_biosynthesis_I) had Q-values under 0.05, likely due to the small sample size (Supplementary Table S3). Captive rhino microbiomes seem to have higher activity for bacterial replication and amino acid production. Additionally, functional pathways and GO terms show indications of higher starch availability in the captive rhinos. The wild and captive rhinos present different pathways, suggesting that different metabolic activity is occurring between the two groups.

## Discussion

The gut microbiome plays a key role in health and the well-being of animals, yet there is no consensus on how the gut microbiome might change between wild and captive animals^4^. In herbivores, the bacterial population in the gut is involved in the breakdown of fibrous plant material into various metabolites including small chain fatty acids (SCFA) that exert a significant impact on host health. Previous studies on gut microbiome diversity in wild compared to captive animals have limited their inference to a single gene for identifying known bacterial taxa, which lacks the genomic breadth and taxonomic depth available through shotgun metagenomics. Here we capitalize on the powerful metagenomics approach to characterize and test for differences in alpha diversity, beta diversity, and functional diversity in the gut (fecal) microbiome of wild and captive black rhinos, a critically endangered species, with the goal of using this information to improve health in captive animals.

Because we collected samples both in the US (captive) and South Africa (wild), we used two different kits (ZymoBIOMICs and MoBio [now Qiagen] PowerFecal) for preservation and DNA extraction due to regional availability of these kits. The Zymo-extracted samples produced more mapped sequencing read results than MoBio-extracted samples, irrelevant of bioinformatic software used (PathoScope, Kraken, Centrifuge). There was a difference in OTU richness between the two extraction methods utilized in this study, and the collection/extraction procedures did influence the microbiome composition. Although alpha diversity (Shannon and Simpson diversity indices) were similar between the two extraction kits, there were distinct differences between Zymo and MoBio extracted samples. This difference could be attributed to differences in the kits used. Specifically, the Zymo kit was designed to efficiently isolate bacterial, fungal, protozoan, algae, viral, mitochondrial, and host DNA from mammalian feces, soil, fungal/bacterial cells, biofilms and water. Thus, the Zymo kit is more generic and therefore was better optimized for broader microbiome usage in contrast to the MoBio kit which was optimized for human fecal samples. Furthermore, the samples extracted using the Zymo kit were stored in Zymo DNA/RNA Shield preservation solution, and thus may have preserved more of the microbes between time of collection to extraction. Therefore, care should be exercised during collection, storage and processing of fecal samples from wildlife for metagenomic analyses, especially under field conditions.

A high proportion of bacteria identified in fecal samples were conserved between wild and captive rhinos but differences that distinguished a significant change in microbial communities due to captivity were also detected, as also seen by several other captivity studies utilizing 16S amplicon sequencing on mammals^5,13–17^. However, our metagenomic approach resulted in a large number of unmappable sequencing reads (~90%) from the black rhinos, suggesting a lack of relevant and known bacterial genomes in the database. One of the great advantages of the metagenomics approach is that you can discover and quantify the unknown microbes as well as the known and our results indicate that targeted 16S amplicon sequencing is missing much of the microbial diversity given that 90% of the reads could not be mapped to reference genomes. This result was validated across three different software platforms for characterizing microbiome diversity, namely PathoScope, Kraken, and Centrifuge. Due to the significant lack of curated and verified microbial genomes from wildlife in genomic databases (i.e., NCBI’s RefSeq), there is a critical need to investigate these under-studied systems (i.e., wildlife and the seasonal dynamics of their diverse microbiomes) to reconstruct new genomes to fully identify the organisms present in their microbiome. Identifying the unknown microbes from wildlife microbiomes will provide the entire research and wildlife health communities with the necessary information to accurately characterize and potentially alter the microbiome of captive species to improve health.

While we found no significant difference in alpha-diversity of microbial communities between wild and captive populations of black rhino (both had high numbers of microbial species), the beta-diversity was significantly different suggesting there are distinct microbial communities in wild versus captive black rhinos. Our results showed increased assignment of microbial reads in the captive samples to bacterial taxa traditionally associated with ruminant guts (such as *Ruminococcus albus*, *Selenomonas bovis*, and *Treponema bryantii*), suggesting that the unknown (prokaryotic genomes not present in NCBI’s RefSeq) gut microbes of wild rhinos were being replaced by those found in conventional human-domesticated livestock. This replacement could be partially due to the rhinos receiving a similar diet to cows and horses or to the humans that captive rhinos are often in contact with. Wild rhinos seem to follow a microbiome profile closer to healthier domestic animals, with greater beta diversity, functional diversity, and variation between individual rhinos compared to captive rhinos^18^.

With our metagenomic data, we were able to establish a core microbiome for both the wild and captive black rhino. The wild black rhinos’ microbiome comprised of Firmicutes (51%), Proteobacteria (23.6%) and Bacteroidetes (17.6%). In contrast, the microbiome of captive black rhinos comprised of Firmicutes (48%) and Bacteroidetes (42.4%). Similarly, an earlier study reported that the white rhinoceros gut microbiome was predominantly comprised of Firmicutes and Bacteroidetes constituting over 90% of total sequences^19^. Interestingly, Firmicutes and Bacteroidetes represented the most ubiquitous taxa in the vertebrate microbiome and Firmicutes was determined to be the most abundant phyla in the gut of healthy humans and other mammals^20^. Our results show, at the phylum level, that captive black rhino microbiome diversity mirrored the white rhino (captive) with a preponderance of Firmicutes and Bacteroidetes^19^. A recent study compared microbiome diversity of captive southern white rhino (*Ceratotherium simum simum*) and captive greater one-horned rhino (*Rhinoceros unicornis*) using 16S sequencing^21^. They, too, found predominantly Firmicutes and Bacteroidetes, consistent with our captive black rhinos, but in different proportions with the southern white rhino having more Bacteroidetes (55%:30%), whereas the greater one-horned rhino had more Firmicutes (55%:33%)^21^. Thus, the wild animals seem to have reduced Bacteroidetes and novel Proteobacteria compared to captive rhinos, and captive rhinos of different species seem to have converged on a dominance of Bacteriodetes and Firmicutes to the exclusion of Proteobacteria.

With very few studies existing on rhino microbiome, their closest domestic relative, horses, can be utilized as a source of comparison. Previous studies in healthy horses have shown that Firmicutes are seen in a higher ratio compared to Bacteroidetes^22^, while higher proportions of Bacteriodetes are associated with colitis^23^. However, there is minimal information on the incidence of colitis in captive rhinos and warrants further investigation. In contrast, Proteobacteria constituted the second most abundant phyla in the wild counterparts. Although Proteobacteria is considered a core microbe of herbivores^24^, this phylum also includes a wide variety of well-known pathogens like *Eschericia coli*, *Salmonella*, *Vibrio*, *Helicobactor* and others^25^. These findings may be influenced by the fact that in the wild rhinos share water sources (water holes) often visited by numerous other species. It is not uncommon that animals defecate in these areas and as a result, contaminate the water with various other microbes that in turn could establish in the gut of animals consuming this water.

A comparison of the functional diversity in the black rhino microbiome demonstrated a greater abundance of glycolysis and amino acid synthesis pathways in captive compared to their wild counterparts suggesting dysbiosis resulting from diet offered in captivity. Captive black rhinos also showed indications of high starch availability. Captive rhino diets consist of ~36% neutral detergent fiber (NDF) and ~25% acid detergent fiber (ADF) in the commercial products, 36–50% NDF and 28–39% ADF in alfalfa hay, and 49–69% NDF and 31–41% ADF in grass hay^26^. The largest proportion of the diet comes from alfalfa hay and commercial products, which represents a lower fiber content range than what wild rhinos have been observed comsuming with NDF ranging from 30–78% and ADF ranging from 14–59%^27^. As such, when compared with their captive counterparts, the microbiome of wild black rhinos contained a higher proportion of bacteria involved in breakdown of plant materials. Specifically, we identified higher proportions of *Escherichia*, *Oscillibacter*, *Pseudobutyrivibrio*, and *Treponema* in wild black rhinos. All these taxa are known to be involved in breakdown of fibers. Furthermore, *Pseudobutyrivibrio* are involved in butyrate production, which has also been reported to be higher in healthy animals by supporting healthier papillae in the gut^28^. The SCFAs acetate, butyrate and propionate are important in several physiological aspects of the host’s nutrient acquisition, immune function, cell signaling, and pathogen protection^29^.

Our study represents the most extensive analysis of the gut microbiome of free-ranging (wild) southern black rhinoceros capitalizing on the more powerful and insightful metagenomic sequencing approach. Similar to earlier studies in other large herbivores, we identified a core microbiome comprising of Firmicutes, Bacteriodetes, and Proteobacteria in the wild black rhinos. These phyla have been reported in most hindgut fermenters and are involved in breakdown of fibrous plant material (polysaccharides). Comparison of gut microbiome between wild and captive rhinos demonstrates a preponderance of bacterial families involved in carbohydrate metabolism. Although this is preliminary, the physiological significance of this new finding cannot be overlooked. Several studies have shown that increased utilization of carbohydrates could lead to dysbiosis in the gut and associated changes in systemic immune function. However, further analysis of a large population of captive managed black rhinos would help confirm these findings and also examine the impact, if any, on metabolic status and immune function. The metagenomic sequencing provides a new minimally invasive and high resolution technique for evaluating nutrition and response to potential interventions. Given the differences discovered between the wild and captive gut microbiomes of the black rhino, there is a clear path to potentially altering the captive gut microbiome to better reflect the wild microbiome diversity and test for improved overall health of captive populations. This could be achieved through a combination of changes in captive black rhino diet, administration of probiotics to better reflect the wild rhino core microbiome, and/or the application of a fecal transplant to restore gut microbiome diversity^30^. Future studies should sample wild and captive rhinos longitudinally to assess temporal and seasonal variation in the gut microbiome to better inform approaches to restore a healthly microbiome in captive populations.

## Materials and Methods

### Animal use statement

All animals were opportunistically sampled during routine translocation efforts in South Africa or during routine health assessments in the United States. Hence, no Institutional Animal Care and Use approval was required.

### Collection

Permits to collect, process, and transport samples (both within South Africa and to the US) from wild and captive black rhinos were obtained from the South African National Parks, CITES, and US Fish and Wildlife service. A total of 25 fecal samples were collected from 17 wild and 8 captive black rhinos (Table 2). All wild animals were opportunistically sampled during routine translocation efforts in South Africa. Animals were immobilized using a combination of etorphine (9.8 mg/ml, Novartis, Kempton Park 1619, South Africa), azaperone (40 mg/ml, Janssen Pharmaceutical Ltd., Halfway House 1685, South Africa) and hyaluronidase (5000 i.u./vial, Kyron Laboratories, Benrose 2011, South Africa) delivered remotely by dart. At the end of the procedure, naltrexone (40 mg/ml, Kyron laboratories) was administered intravenously to reverse the immobilization. All wild rhinos were considered healthy based on physical appearance and behavior and received no supplemental commercial diets. The eight captive samples were collected from black rhinos located on a private ranch in Texas as well as an Association of Zoos and Aquariums accredited institution (also in Texas). Captive animals were fed Alfalfa hay (2 squares), coastal grass hay (1 square), a grain-free hay enhancer (Elephant and White Rhino Supplement, Mazuri, St. Louis, MO; 5 lbs), Strategy healthy edge (a high-fat nuggets that delivers a controlled starch and sugar as well as higher fat and fiber; Purina Animal Nutrition, Gary Summit, MO; 5 lbs), a stabilized rice bran supplement (Max-E-, MannaPro, Chesterfiled, MO; 1 lb), and fresh cut huisache twice daily. Animals also received electrolyte powder (Electro Dex; Farnam Companies Inc; Phoenix, AZ; 2 oz per 20 gallons of water daily) as well as apples and sweet potatoes for treats.

**Table 2:** List of all black rhinos sampled with corresponding metadata and captivity status.

Sample Number	Captivity Status	Extraction Kit	DNA/RNA Shield	Sex	Age	Sample Type
R01	Wild	MoBio	No	M	SAd	Feces
R02	Wild	MoBio	No	F	SAd	Feces
R03	Wild	MoBio	No	M	Juvenile	Feces
R04	Wild	MoBio	No	F	SAd	Feces
R05	Wild	MoBio	No	F	SAd	Feces
R06	Wild	MoBio	No	M	SAd	Feces
R07	Wild	MoBio	No	F	Ad	Feces
R08	Wild	MoBio	No	F	Ad	Feces
R11	Wild	Zymo	Yes	M	Ad	Feces
R12	Wild	Zymo	Yes	M	Ad	Feces
R14	Wild	Zymo	Yes	M	Ad	Feces
R15	Wild	Zymo	Yes	M	Ad	Feces
R16	Wild	Zymo	Yes	F	SAd	Feces
R17	Wild	Zymo	Yes	M	Ad	Feces
R18	Wild	Zymo	Yes	F	SAd	Feces
R19	Wild	Zymo	Yes	M	Juvenile	Feces
R20	Wild	Zymo	Yes	M	Ad	Feces
R21	Captive	Zymo	Yes	F	Ad	Feces
R22	Captive	Zymo	Yes	M	Ad	Feces
R23	Captive	Zymo	Yes	F	Ad	Feces
R24	Captive	Zymo	Yes	M	Ad	Feces
R25	Captive	Zymo	Yes	F	Ad	Feces
R26	Captive	Zymo	Yes	F	Ad	Feces
R27	Captive	Zymo	Yes	F	Ad	Feces
R28	Captive	Zymo	Yes	F	Ad	Feces

Abbreviations: adult (Ad), senior adult (Sad), male (M), female (F), MoBio PowerFecal kit, which is now QIAamp PowerFecal DNA kit (MoBio), and ZymoBIOMICS DNA Miniprep (Zymo).

The eight captive fecal samples were stored frozen (−80 °C) until DNA extraction. Eight of the 17 wild samples were transported to Stellenbosch University in South Africa for DNA extraction and shipped to the United States as purified genomic DNA. For the remaining nine wild fecal samples, between 1–2 grams of feces were stored in DNA/RNA Shield (Zymo Research, USA) and transported into the United States. These samples also were stored frozen (−80 °C) until DNA extraction was attempted.

### DNA extraction and metagenomic sequencing

Fecal samples R1-R8 (wild rhinos) were processed for DNA extraction in South Africa using the MoBio PowerFecal kit (now QIAamp PowerFecal DNA kit; QIAGEN, USA) per manufacturer’s instructions. Samples (R11-28; consisting of wild and captive rhinos) stored in DNA/RNA Shield were processed (ZymoBIOMICS DNA Miniprep extraction kit; Zymo Research, USA) per manufacturer’s instructions. Different extraction kits were used due to the different locations and availability of kits for the molecular work. In order to minimize biases from extraction method, we placed a 1g scoop of feces from each captive sample into the Zymo Research DNA/RNA Shield resulting in 1 mL of fresh fecal material in solution and were stored frozen until processing (as described above). The only alterations to the Zymo extraction kit manufacturer’s instructions were the following: 1) we began with the sample amount of 1 mL of DNA/RNA Shield that contained the fecal sample and 2) we secured the samples in a bead beater and processed at maximum speed for 30 minutes. All DNA samples were processed for sequencing using an Illumina Nextera XT library prep kit (Illumina, Inc., USA) and then sequenced with a single High Output v2 Kit (300 cycles) run on an Illumina NextSeq 500 platform (Illumina, Inc., USA) at the George Washington University Milken Institute School of Public Health Genomics Core Facility. We compared the number of OTUs identified, observed species richness, Simpson and Shannon diversity indices, and dissimilarity metrics between wild samples extracted with the MoBio extraction kit and the Zymo extraction kit to test for biases associated with extraction approaches.

### Bioinformatic analyses

Quality of the reads was assessed using FastQC v. 0.11.5 (https://www.bioinformatics.babraham.ac.uk/projects/fastqc/). Short reads, low quality reads, and reads with adapter contamination and low quality bases were removed from the FASTQ files by trimming using Flexbar v. 3.0.3^31^. Then low complexity reads were removed from analysis with PRINSEQ v. 0.20.4^32^. Following data quality check, FastQC was repeated to assess the efficacy of quality trimming and cleaning. The resulting high quality reads were mapped with PathoScope 2.0’s^33^ mapping module^34^, which utilizes a wrapper for Bowtie2^35^, to the representative and reference prokaryote, viroid, and virus genome databases available from GenBank (https://www.ncbi.nlm.nih.gov/genbank/). Reads that mapped best to the rhino genome (white rhinoceros: *Ceratotherium simum*, NCBI Assembly ID: 406328), human genome (hg38), plant genomes from Gramene (http://www.gramene.org), the representative and reference genomes databases for fungi and protozoan from GenBank or to the WormBase parasite genome database (http://parasite.wormbase.org/index.html) also were removed. We assessed levels of contamination in the sequencing reads by the number of reads that mapped better to any database but the prokaryotic database. PathoScope 2.0 ID module^33^ was used to assign taxonomy. We also generated taxonomic assignments using PhyloSift’s v. 1.0.1 core and extended marker sets v. 1413946442, which phylogenetically places reads matching conserved marker genes to infer taxonomy^36^. We utilized additional metagenomic sequence classification software Centrifuge v. 1.0.3-beta^37^ and Kraken v. v0.10.5-beta^38^ to assign taxonomy and validate PhyloSift and PathosSope results. Functional analysis was completed using HUMAnN2 with the full UniRef90 database (http://huttenhower.sph.harvard.edu/humann2)^39^. Subsequently, HUMAnN2 results identified pathways and were grouped into GO terms and tested for associations with captivity status using MaAsLin: Multivariate Association with Linear Models^40^ and filtered with a q-value of 0.05.

We analyzed and visualized the PhyloSift and PathoScope with R^41^ v. 3.5.0 in Rstudio^42^ v. 1.1.453 using the phyloseq^43^, vegan^44^, DESeq2^45^, plotly^46^ and ggplot2^47^ packages. For taxonomic composition visualizations, OTUs were transformed into relative abundances and then filtered to include only microbes that had a mean above 1% or a maximum prevalence in any sample greater than or equal to 5%. The core microbiome for each wild and captive rhinos was defined as those that were present in at least 50% of the samples with greater than 0.1% relative abundance^13^. Differential abundance analysis between all wild and captive rhinos was conducted with the DESeq2 and Phyloseq packages with the PathoScope data. Data (OTU counts) were log-transformed and variance-stabilized using geometric means to normalize sequencing depth across samples. We determined significant species with an 0.01 alpha level that we then used to filter the adjusted p-value; additional filtering based on relative abundance was not completed. Observed species richness, Shannon diversity index, and Simpson diversity index, which reflect the richness and evenness of microbial representation in a sample, were estimated using the phyloseq and DESeq2 R packages. ANOVA and Kruskal-Wallis tests in R were applied to compare extraction kit differences between the wild microbiome samples and between wild versus captive microbiomes. Alpha diversity metrics were analyzed with lmerTest^48^ R package. The linear mixed-effects (LME) model analysis was implemented to test for associations between alpha diversity indices and taxa abundances, extraction kit, age and sex. Analysis showed that the only co-variable that showed an impact on the representation of microbial analyses was the extraction kit, and therefore age and sex were not used in final diversity analyses. Additionally, Jaccard (presence-absence) and Bray-Curtis (abundance-weighted) indices were used and implemented in the vegan R package to calculate the similarity of microbial communities between samples using OTU matrices generated from the PathoScope output files. The resulting distance matrices, non-metric multidimensional scaling (NMDS) and calculated dissimilarity metrics with Bray-Curtis, Jaccard, and JSD indices, were compared with permutational multivariate analysis of variance (PERMANOVA)^49^ with the vegan^44^ R package and significance was determined with 10,000 permutations.

## Supplementary information

Supplementary information

## Data Availability

The next-generation sequencing data assocatied with this study have been deposited in GenBank under SRA accession: PRJNA532626.
