# Supplementary material for: Gut microbiome differences between wild and captive black rhinoceros – implications for rhino health
Source: Sci Rep. 2019 May 28;9:7570. doi: 10.1038/s41598-019-43875-3 (PMC6538756; doi:10.1038/s41598-019-43875-3)
Supplement: Supplementary file 1 — Supplementary information [file 41598_2019_43875_MOESM1_ESM.pdf]

Gut microbiome differences between wild and captive black rhinoceros – implications for rhino health

Keylie M. Gibson[1,2], Bryan N. Nguyen[1,2], Laura M. Neumann[3], Michele Miller[4], Peter Buss[5], Savel Daniels[6], Michelle Ahn[1,2], Keith A. Crandall[1,7*], & Budhan Pukazhenthi[8*]

1 Computational Biology Institute, The Milken Institute School of Public Health, George Washington University, Washington, DC, USA
2 Department of Biological Sciences, George Washington University, Washington, DC, USA
3 Department of Environmental and Occupational Health, The Milken Institute School of Public Health, The George Washington University, Washington, DC, USA
4 DST-NRF Centre of Excellence for Biomedical Tuberculosis Research; South African Medical Research Council Centre for Tuberculosis Research; Division of Molecular Biology and Human Genetics, Faculty of Medicine and Health Sciences, Stellenbosch University, Cape Town, South Africa.
5 South African National Parks, Veterinary Wildlife Services, Kruger National Park, Skukuza, South Africa
6 Department of Botany and Zoology, University of Stellenbosch, Private Bag X1, Matieland 7602, South Africa
7 Department of Epidemiology and Biostatistics, The Milken Institute School of Public Health, George Washington University, Washington, DC, USA
8 Smithsonian's National Zoo and Conservation Biology Institute, Front Royal, VA, USA
* Co-Senior Authors

Corresponding author:
Budhan Pukazhenthi
Smithsonian Conservation Biology Institute
1500 Remount Road, Front Royal, VA 22630
pukazhenthib@si.edu

Supplemental table legends

Table S1a. Core rhino microbiome species in wild rhinos.

Table S1b. Core rhino microbiome species in captive rhinos.

Table S2. Differentially gene ontology terms between wild and captive rhino samples.

Table S3. Differentially abundant pathways between wild and captive rhino samples.

Table S1a. Core rhino microbiome species in wild rhinos.

TaxID	superkingdom	phylum	class	order	family	genus	species
1034345	Bacteria	Actinobacteria	Coriobacteriia	Coriobacteriales	Coriobacteriaceae	Senegalimassilia	Senegalimassilia anaerobia
1073376	Bacteria	Firmicutes	Clostridia	Clostridiales	Ruminococcaceae	Ruminococcus	Ruminococcus lactaris
1120998	Bacteria	Firmicutes	Clostridia	Clostridiales	Clostridiales Family XIII. Incertae Sedis	Anaerovorax	Anaerovorax odorimutans
1121115	Bacteria	Firmicutes	Clostridia	Clostridiales	Lachnospiraceae	Blautia	Blautia wexlerae
1121334	Bacteria	Firmicutes	Clostridia	Clostridiales	Ruminococcaceae	Ruminiclostridium	[Clostridium] sporosphaeroides
1122230	Archaea	Euryarchaeota	Methanomicrobia	Methanomicrobiales	Methanocorpusculaceae	Methanocorpusculum	Methanocorpusculum bavaricum
1122978	Bacteria	Bacteroidetes	Bacteroidia	Bacteroidales	Prevotellaceae	Prevotella	Prevotella albensis
1203606	Bacteria	Firmicutes	Clostridia	Clostridiales	Clostridiaceae	Butyricicoccus	Butyricicoccus pullicaecorum
1236689	Archaea	Euryarchaeota	Thermoplasmata	Methanomassiliicoccales	Methanomassiliicoccaceae	Candidatus Methanomethylophilus	Candidatus Methanomethylophilus alvus
1256908	Bacteria	Firmicutes	Clostridia	Clostridiales	Eubacteriaceae	Eubacterium	Eubacterium ramulus
1321814	Bacteria	Firmicutes	Clostridia	Clostridiales	Clostridiales Family XIII. Incertae Sedis	NA	[Eubacterium] brachy
1384484	Bacteria	Actinobacteria	Coriobacteriia	Eggerthellales	Eggerthellaceae	Adlercreutzia	Adlercreutzia equolifaciens
1401079	Bacteria	Firmicutes	Clostridia	Clostridiales	Clostridiales Family XIII. Incertae Sedis	Mogibacterium	Mogibacterium timidum
1408189	Bacteria	Actinobacteria	Actinobacteria	Corynebacteriales	Corynebacteriaceae	Corynebacterium	Corynebacterium lactis
1408437	Bacteria	Firmicutes	Clostridia	Clostridiales	Ruminococcaceae	Agathobaculum	Agathobaculum desmolans
1458469	Bacteria	Firmicutes	Clostridia	Clostridiales	Lachnospiraceae	Pseudobutyrivibrio	Pseudobutyrivibrio ruminis
264731	Bacteria	Bacteroidetes	Bacteroidia	Bacteroidales	Prevotellaceae	Prevotella	Prevotella ruminicola
267747	Bacteria	Actinobacteria	Actinobacteria	Propionibacteriales	Propionibacteriaceae	Cutibacterium	Cutibacterium acnes
295405	Bacteria	Bacteroidetes	Bacteroidia	Bacteroidales	Bacteroidaceae	Bacteroides	Bacteroides fragilis
411459	Bacteria	Firmicutes	Clostridia	Clostridiales	Lachnospiraceae	Blautia	Blautia obeum
411461	Bacteria	Firmicutes	Clostridia	Clostridiales	Lachnospiraceae	Dorea	Dorea formicigenerans
411463	Bacteria	Firmicutes	Clostridia	Clostridiales	Eubacteriaceae	Eubacterium	Eubacterium ventriosum
411469	Bacteria	Firmicutes	Clostridia	Clostridiales	Eubacteriaceae	Eubacterium	[Eubacterium] hallii
411471	Bacteria	Firmicutes	Clostridia	Clostridiales	Ruminococcaceae	Subdoligranulum	Subdoligranulum variabile
411483	Bacteria	Firmicutes	Clostridia	Clostridiales	Ruminococcaceae	Faecalibacterium	Faecalibacterium prausnitzii
428125	Bacteria	Firmicutes	Clostridia	Clostridiales	Ruminococcaceae	Ruminiclostridium	[Clostridium] leptum
470146	Bacteria	Firmicutes	Clostridia	Clostridiales	Lachnospiraceae	Coprococcus	Coprococcus comes
479437	Bacteria	Actinobacteria	Coriobacteriia	Eggerthellales	Eggerthellaceae	Eggerthella	Eggerthella lenta
511680	Bacteria	Firmicutes	Clostridia	Clostridiales	Lachnospiraceae	Butyrivibrio	Butyrivibrio crossotus
515619	Bacteria	Firmicutes	Clostridia	Clostridiales	Lachnospiraceae	NA	[Eubacterium] rectale
518636	Bacteria	Firmicutes	Clostridia	Clostridiales	Lachnospiraceae	Lachnoclostridium	[Clostridium] asparagiforme
537011	Bacteria	Bacteroidetes	Bacteroidia	Bacteroidales	Prevotellaceae	Prevotella	Prevotella copri
548479	Bacteria	Actinobacteria	Actinobacteria	Actinomycetales	Actinomycetaceae	Mobiluncus	Mobiluncus curtisii
585394	Bacteria	Firmicutes	Clostridia	Clostridiales	Lachnospiraceae	Roseburia	Roseburia hominis
59374	Bacteria	Fibrobacteres	Fibrobacteria	Fibrobacterales	Fibrobacteraceae	Fibrobacter	Fibrobacter succinogenes
626522	Bacteria	Bacteroidetes	Bacteroidia	Bacteroidales	Prevotellaceae	Alloprevotella	Alloprevotella tannerae
626939	Bacteria	Firmicutes	Negativicutes	Acidaminococcales	Acidaminococcaceae	Phascolarctobacterium	Phascolarctobacterium succinatutens
641112	Bacteria	Firmicutes	Clostridia	Clostridiales	Ruminococcaceae	Ruminococcus	Ruminococcus flavefaciens
679192	Bacteria	Firmicutes	Erysipelotrichia	Erysipelotrichales	Erysipelotrichaceae	Bulleidia	Bulleidia extructa
685038	Bacteria	Proteobacteria	Gammaproteobacteria	Enterobacterales	Enterobacteriaceae	Escherichia	Escherichia coli
702438	Bacteria	Bacteroidetes	Bacteroidia	Bacteroidales	Prevotellaceae	Prevotella	Prevotella oulorum
706433	Bacteria	Firmicutes	Erysipelotrichia	Erysipelotrichales	Erysipelotrichaceae	Solobacterium	Solobacterium moorei
866499	Bacteria	Synergistetes	Synergistia	Synergistales	Synergistaceae	Cloacibacillus	Cloacibacillus evryensis
879243	Bacteria	Bacteroidetes	Bacteroidia	Bacteroidales	Porphyromonadaceae	Porphyromonas	Porphyromonas asaccharolytica
927704	Bacteria	Firmicutes	Negativicutes	Selenomonadales	Selenomonadaceae	Selenomonas	Selenomonas ruminantium
981539	Bacteria	Firmicutes	Bacilli	Lactobacillales	Streptococcaceae	Streptococcus	Streptococcus gallolyticus
997894	Bacteria	Firmicutes	Clostridia	Clostridiales	Lachnospiraceae	Lachnoclostridium	[Clostridium] bolteae
999412	Bacteria	Firmicutes	Clostridia	Clostridiales	Clostridiaceae	Hungatella	Hungatella hathewayi

Table S1b. Core rhino microbiome species in captive rhinos.

TaxID	superkingdom	phylum	class	order	family	genus	species
1002367	Bacteria	Bacteroidetes	Bacteroidia	Bacteroidales	Prevotellaceae	Prevotella	Prevotella stercorea
1007096	Bacteria	Firmicutes	Clostridia	Clostridiales	Oscillospiraceae	Oscillibacter	Oscillibacter ruminantium
1034345	Bacteria	Actinobacteria	Coriobacteriia	Coriobacteriales	Coriobacteriaceae	Senegalimassilia	Senegalimassilia anaerobia
1121094	Bacteria	Bacteroidetes	Bacteroidia	Bacteroidales	Bacteroidaceae	Bacteroides	Bacteroides barnesiae
1121115	Bacteria	Firmicutes	Clostridia	Clostridiales	Lachnospiraceae	Blautia	Blautia wexlerae
1121296	Bacteria	Firmicutes	Clostridia	Clostridiales	Lachnospiraceae	Lachnoclostridium	[Clostridium] aminophilum
1121334	Bacteria	Firmicutes	Clostridia	Clostridiales	Ruminococcaceae	Ruminiclostridium	[Clostridium] sporosphaeroides
1122975	Bacteria	Bacteroidetes	Bacteroidia	Bacteroidales	Porphyromonadaceae	Porphyromonas	Porphyromonas somerae
1122978	Bacteria	Bacteroidetes	Bacteroidia	Bacteroidales	Prevotellaceae	Prevotella	Prevotella albensis
1122980	Bacteria	Bacteroidetes	Bacteroidia	Bacteroidales	Prevotellaceae	Prevotella	Prevotella baroniae
1123250	Bacteria	Firmicutes	Negativicutes	Selenomonadales	Selenomonadaceae	Selenomonas	Selenomonas bovis
1130798	Bacteria	Firmicutes	Bacilli	Lactobacillales	Lactobacillaceae	Lactobacillus	Lactobacillus mucosae
1203606	Bacteria	Firmicutes	Clostridia	Clostridiales	Clostridiaceae	Butyricicoccus	Butyricicoccus pullicaecorum
1235802	Bacteria	Firmicutes	Clostridia	Clostridiales	Eubacteriaceae	Eubacterium	Eubacterium plexicaudatum
1236509	Bacteria	Bacteroidetes	Bacteroidia	Bacteroidales	Prevotellaceae	Prevotella	Prevotella dentasini
1256908	Bacteria	Firmicutes	Clostridia	Clostridiales	Eubacteriaceae	Eubacterium	Eubacterium ramulus
1280705	Bacteria	Bacteroidetes	Bacteroidia	Bacteroidales	Prevotellaceae	Prevotella	Prevotella bryantii
1294025	Bacteria	Firmicutes	Clostridia	Clostridiales	Lachnospiraceae	Cellulosilyticum	Cellulosilyticum ruminicola
1321814	Bacteria	Firmicutes	Clostridia	Clostridiales	Clostridiales Family XIII. Incertae Sedis	NA	[Eubacterium] brachy
1401079	Bacteria	Firmicutes	Clostridia	Clostridiales	Clostridiales Family XIII. Incertae Sedis	Mogibacterium	Mogibacterium timidum
1408437	Bacteria	Firmicutes	Clostridia	Clostridiales	Ruminococcaceae	Agathobaculum	Agathobaculum desmolans
1408472	Bacteria	Bacteroidetes	Bacteroidia	Bacteroidales	Prevotellaceae	Prevotella	Prevotella brevis
1410649	Bacteria	Firmicutes	Clostridia	Clostridiales	Lachnospiraceae	Blautia	Blautia schinkii
1415626	Archaea	Euryarchaeota	Methanobacteria	Methanobacteriales	Methanobacteriaceae	Methanobrevibacter	Methanobrevibacter oralis
1423740	Bacteria	Firmicutes	Bacilli	Lactobacillales	Lactobacillaceae	Lactobacillus	Lactobacillus equi
1458469	Bacteria	Firmicutes	Clostridia	Clostridiales	Lachnospiraceae	Pseudobutyrivibrio	Pseudobutyrivibrio ruminis
213810	Bacteria	Firmicutes	Clostridia	Clostridiales	Ruminococcaceae	Ruminococcus	Ruminococcus champanellensis
264731	Bacteria	Bacteroidetes	Bacteroidia	Bacteroidales	Prevotellaceae	Prevotella	Prevotella ruminicola
411459	Bacteria	Firmicutes	Clostridia	Clostridiales	Lachnospiraceae	Blautia	Blautia obeum
411463	Bacteria	Firmicutes	Clostridia	Clostridiales	Eubacteriaceae	Eubacterium	Eubacterium ventriosum
411467	Bacteria	Firmicutes	Clostridia	Clostridiales	NA	Pseudoflavonifractor	Pseudoflavonifractor capillosus
411471	Bacteria	Firmicutes	Clostridia	Clostridiales	Ruminococcaceae	Subdoligranulum	Subdoligranulum variabile
411473	Bacteria	Firmicutes	Clostridia	Clostridiales	Ruminococcaceae	Ruminococcus	Ruminococcus callidus
411474	Bacteria	Firmicutes	Clostridia	Clostridiales	Lachnospiraceae	Coprococcus	Coprococcus eutactus
411483	Bacteria	Firmicutes	Clostridia	Clostridiales	Ruminococcaceae	Faecalibacterium	Faecalibacterium prausnitzii
428125	Bacteria	Firmicutes	Clostridia	Clostridiales	Ruminococcaceae	Ruminiclostridium	[Clostridium] leptum
445972	Bacteria	Firmicutes	Clostridia	Clostridiales	Ruminococcaceae	Anaerotruncus	Anaerotruncus colihominis
470146	Bacteria	Firmicutes	Clostridia	Clostridiales	Lachnospiraceae	Coprococcus	Coprococcus comes
471855	Bacteria	Actinobacteria	Coriobacteriia	Eggerthellales	Eggerthellaceae	Slackia	Slackia heliotrinireducens
483218	Bacteria	Firmicutes	Clostridia	Clostridiales	NA	NA	[Bacteroides] pectinophilus

515619	Bacteria	Firmicutes	Clostridia	Clostridiales	Lachnospiraceae		NA	[Eubacterium] rectale
518636	Bacteria	Firmicutes	Clostridia	Clostridiales	Lachnospiraceae		Lachnoclostridium	[Clostridium] asparagiforme
536231	Bacteria	Firmicutes	Clostridia	Clostridiales	Lachnospiraceae		Roseburia	Roseburia intestinalis
537011	Bacteria	Bacteroidetes	Bacteroidia	Bacteroidales	Prevotellaceae		Prevotella	Prevotella copri
547042	Bacteria	Bacteroidetes	Bacteroidia	Bacteroidales	Bacteroidaceae		Bacteroides	Bacteroides coprophilus
585394	Bacteria	Firmicutes	Clostridia	Clostridiales	Lachnospiraceae		Roseburia	Roseburia hominis
59374	Bacteria	Fibrobacteres	Fibrobacteria	Fibrobacterales	Fibrobacteraceae		Fibrobacter	Fibrobacter succinogenes
608534	Bacteria	Firmicutes	Clostridia	Clostridiales	Lachnospiraceae		Oribacterium	Oribacterium sp. oral taxon 078
622312	Bacteria	Firmicutes	Clostridia	Clostridiales	Lachnospiraceae		Roseburia	Roseburia inulinivorans
626939	Bacteria	Firmicutes	Negativicutes	Acidaminococcales	Acidaminococcaceae		Phascolarctobacterium	Phascolarctobacterium succinatutens
633697	Bacteria	Firmicutes	Clostridia	Clostridiales	Eubacteriaceae		Eubacterium	[Eubacterium] cellulosolvens
634498	Archaea	Euryarchaeota	Methanobacteria	Methanobacteriales	Methanobacteriaceae		Methanobrevibacter	Methanobrevibacter ruminantium
641112	Bacteria	Firmicutes	Clostridia	Clostridiales	Ruminococcaceae		Ruminococcus	Ruminococcus flavefaciens
679190	Bacteria	Bacteroidetes	Bacteroidia	Bacteroidales	Prevotellaceae		Prevotella	Prevotella buccalis
697329	Bacteria	Firmicutes	Clostridia	Clostridiales	Ruminococcaceae		Ruminococcus	Ruminococcus albus
717959	Bacteria	Bacteroidetes	Bacteroidia	Bacteroidales	Rikenellaceae		Alistipes	Alistipes shahii
742733	Bacteria	Firmicutes	Clostridia	Clostridiales	Lachnospiraceae		Lachnoclostridium	[Clostridium] citroniae
742742	Bacteria	Actinobacteria	Coriobacteriia	Coriobacteriales	Coriobacteriaceae		Collinsella	Collinsella tanakaei
763034	Bacteria	Bacteroidetes	Bacteroidia	Bacteroidales	Bacteroidaceae		Bacteroides	Bacteroides fluxus
869209	Bacteria	Spirochaetes	Spirochaetia	Spirochaetales	Spirochaetaceae		Treponema	Treponema succinifaciens
877418	Bacteria	Spirochaetes	Spirochaetia	Spirochaetales	Spirochaetaceae		Treponema	Treponema bryantii
927704	Bacteria	Firmicutes	Negativicutes	Selenomonadales	Selenomonadaceae		Selenomonas	Selenomonas ruminantium
947969	Bacteria	Actinobacteria	Actinobacteria	Micrococcales	Cellulomonadaceae		Cellulomonas	Cellulomonas carbonis
997894	Bacteria	Firmicutes	Clostridia	Clostridiales	Lachnospiraceae		Lachnoclostridium	[Clostridium] bolteae
999419	Bacteria	Bacteroidetes	Bacteroidia	Bacteroidales	Tannerellaceae		Parabacteroides	Parabacteroides johnsonii

Table S2. Differentially gene ontology terms between wild and captive rhino samples.

GOterm	GOname	Taxonomy	Coefficient	Greater_in	N	N not 0	P-value	Q-value
4803	transposase activity	cumulative	-0.018977503	captive	25	25	5.95E-08	5.30E-06
6313	transposition, DNA-mediated	cumulative	-0.018905436	captive	25	25	7.59E-08	5.64E-06
3924	GTPase activity	cumulative	-0.003860804	captive	25	25	9.35E-06	2.98E-04
5525	GTP binding	cumulative	-0.004824809	captive	25	25	2.03E-05	5.67E-04
8170	N-methyltransferase activity	cumulative	-0.004518003	captive	25	25	2.40E-05	6.29E-04
150	recombinase activity	cumulative	-0.007088853	captive	25	25	3.43E-05	8.06E-04
9307	DNA restriction-modification system	cumulative	-0.003276179	captive	25	25	7.37E-05	0.001184718
6306	DNA methylation	cumulative	-0.003897461	captive	25	25	7.75E-05	0.001184718
4386	helicase activity	cumulative	-0.003163252	captive	25	25	1.40E-04	0.001681725
3677	DNA binding	cumulative	-0.017890208	captive	25	25	1.47E-04	0.001684913
9291	unidirectional conjugation	cumulative	-0.003344105	captive	25	25	3.74E-04	0.003553688
6352	DNA-templated transcription, initiation	cumulative	-0.004446882	captive	25	25	4.99E-04	0.004190296
6310	DNA recombination	cumulative	-0.005737998	captive	25	25	5.07E-04	0.004190296
71973	bacterial-type flagellum-dependent cell motility	cumulative	0.003057348	wild	25	25	6.88E-04	0.005288794
15074	DNA integration	cumulative	-0.006468408	captive	25	25	8.65E-04	0.006323693
10181	FMN binding	cumulative	-0.003589327	captive	25	25	9.23E-04	0.006641177
43565	sequence-specific DNA binding	cumulative	-0.007122494	captive	25	25	9.50E-04	0.006726295
271	polysaccharide biosynthetic process	cumulative	-0.001754949	captive	25	25	0.001110279	0.007606796
3887	DNA-directed DNA polymerase activity	cumulative	-0.003117624	captive	25	25	0.001323953	0.008557724
8080	N-acetyltransferase activity	cumulative	-0.005073349	captive	25	25	0.001426879	0.008963215
16987	bacterial sigma factor activity	cumulative	-0.003653918	captive	25	25	0.001557721	0.009649219
4553	hydrolase activity, hydrolyzing O-glycosyl compounds	cumulative	-0.006255836	captive	25	25	0.001715678	0.010224798
NA	various ungrouped proteins	cumulative	0.033255606	wild	25	25	0.002575304	0.014725455
6261	DNA-dependent DNA replication	cumulative	-0.001656474	captive	25	25	0.002743521	0.015295129
6730	one-carbon metabolic process	cumulative	-0.001359434	captive	25	25	0.003606838	0.01940042
3684	damaged DNA binding	cumulative	-0.002134759	captive	25	25	0.003765642	0.019993766
3824	catalytic activity	cumulative	-0.004506958	captive	25	25	0.004315643	0.022547667
31419	cobalamin binding	cumulative	-0.002673432	captive	25	25	0.004397261	0.022547667
6520	cellular amino acid metabolic process	cumulative	-0.001537363	captive	25	25	0.004398312	0.022547667
16639	oxidoreductase activity, acting on the CH-NH2 group of donors, NAD or NADP as acceptor	cumulative	-0.001231881	captive	25	25	0.004775044	0.023662998
4519	endonuclease activity	cumulative	-0.002798129	captive	25	25	0.005385287	0.026106934
16903	oxidoreductase activity, acting on the aldehyde or oxo group of donors	cumulative	-0.001309714	captive	25	25	0.005552445	0.026344579
NA	unmapped reads	cumulative	-0.021814684	captive	25	25	0.006207514	0.029142642
55085	transmembrane transport	cumulative	-0.003221919	captive	25	25	0.006577364	0.030557338
16020	membrane	cumulative	-0.003198458	captive	25	25	0.007610272	0.034788958
5887	integral component of plasma membrane	cumulative	-0.001367803	captive	25	25	0.00764421	0.034788958
3700	DNA binding transcription factor activity	cumulative	-0.005481136	captive	25	25	0.009388698	0.0391342
6047	UDP-N-acetylglucosamine metabolic process	cumulative	-0.001535472	captive	25	25	0.009704007	0.039817305
NA	various ungrouped proteins	g__Erysipelotrichaceae_noname_s__Erysipelotrichaceae_bacterium_5_2_54FAA	-0.006510289	captive	25	6	5.71E-04	0.004464906
5886	plasma membrane	g__Escherichia_s__Escherichia_coli	0.001528117	wild	25	9	0.008516832	0.036117758
5524	ATP binding	g__Escherichia_s__Escherichia_coli	0.001150154	wild	25	9	0.008519472	0.036117758
6351	transcription, DNA-templated	g__Escherichia_s__Escherichia_coli	0.001006386	wild	25	9	0.008521457	0.036117758
3677	DNA binding	g__Escherichia_s__Escherichia_coli	0.001076716	wild	25	9	0.008554774	0.036117758
5737	cytoplasm	g__Escherichia_s__Escherichia_coli	0.001506239	wild	25	9	0.008584041	0.036117758
5975	carbohydrate metabolic process	g__Prevotella_s__Prevotella_ruminicola	-0.008453279	captive	25	10	3.15E-05	7.80E-04
5840	ribosome	g__Prevotella_s__Prevotella_ruminicola	-0.009611483	captive	25	10	4.00E-05	8.81E-04
6412	translation	g__Prevotella_s__Prevotella_ruminicola	-0.011424712	captive	25	10	4.23E-05	8.81E-04
3735	structural constituent of ribosome	g__Prevotella_s__Prevotella_ruminicola	-0.011342597	captive	25	10	4.35E-05	8.81E-04
19843	rRNA binding	g__Prevotella_s__Prevotella_ruminicola	-0.008945235	captive	25	10	4.74E-05	9.18E-04
5886	plasma membrane	g__Prevotella_s__Prevotella_ruminicola	-0.001486209	captive	25	10	1.10E-04	0.001439447
5524	ATP binding	g__Prevotella_s__Prevotella_ruminicola	-0.002373905	captive	25	10	1.25E-04	0.001592247
46872	metal ion binding	g__Prevotella_s__Prevotella_ruminicola	-0.00150565	captive	25	10	1.29E-04	0.001596675
5737	cytoplasm	g__Prevotella_s__Prevotella_ruminicola	-0.002779891	captive	25	10	1.43E-04	0.001681725
3677	DNA binding	g__Prevotella_s__Prevotella_ruminicola	-0.001449915	captive	25	10	2.88E-04	0.002897769
16021	integral component of membrane	g__Prevotella_s__Prevotella_ruminicola	-0.002342284	captive	25	10	2.97E-04	0.002897769
NA	various ungrouped proteins	g__Prevotella_s__Prevotella_ruminicola	-0.003609818	captive	25	10	2.99E-04	0.002897769

	16021	integral component of membrane	g__Ruminococcus_s__Ruminococcus_albus	-0.00696066	captive	25	7	8.52E-05	0.001187059
NA		various ungrouped proteins	g__Ruminococcus_s__Ruminococcus_albus	-0.010977811	captive	25	7	2.87E-04	0.002897769
NA		various ungrouped proteins	g__Ruminococcus_s__Ruminococcus_flavefaciens	-0.022380325	captive	25	15	2.60E-04	0.002756452
	6352	DNA-templated transcription, initiation	unclassified	-0.00410299	captive	25	25	8.27E-09	2.02E-06
	4803	transposase activity	unclassified	-0.019633513	captive	25	25	1.25E-08	2.02E-06
	6313	transposition, DNA-mediated	unclassified	-0.01964708	captive	25	25	1.36E-08	2.02E-06
	16987	bacterial sigma factor activity	unclassified	-0.003592461	captive	25	25	1.97E-08	2.19E-06
	43565	sequence-specific DNA binding	unclassified	-0.007768412	captive	25	25	1.30E-07	8.26E-06
	5525	GTP binding	unclassified	-0.004373969	captive	25	25	5.15E-07	2.87E-05
	10181	FMN binding	unclassified	-0.003603308	captive	25	25	5.86E-07	2.90E-05
	3677	DNA binding	unclassified	-0.018799341	captive	25	25	1.80E-06	8.04E-05
	3924	GTPase activity	unclassified	-0.003172322	captive	25	25	2.34E-06	9.48E-05
	3700	DNA binding transcription factor activity	unclassified	-0.005645729	captive	25	25	2.65E-06	9.86E-05
	6310	DNA recombination	unclassified	-0.006046712	captive	25	25	4.32E-06	1.48E-04
	8170	N-methyltransferase activity	unclassified	-0.004413655	captive	25	25	1.87E-05	5.57E-04
	150	recombinase activity	unclassified	-0.006233922	captive	25	25	5.86E-05	0.001089815
	4519	endonuclease activity	unclassified	-0.003274451	captive	25	25	6.34E-05	0.001126164
	9307	DNA restriction-modification system	unclassified	-0.003243699	captive	25	25	6.57E-05	0.001126164
	16787	hydrolase activity	unclassified	-0.005106196	captive	25	25	7.63E-05	0.001184718
	6306	DNA methylation	unclassified	-0.003758307	captive	25	25	7.97E-05	0.001184718
	4386	helicase activity	unclassified	-0.00311011	captive	25	25	8.33E-05	0.001187059
	271	polysaccharide biosynthetic process	unclassified	-0.00183968	captive	25	25	9.03E-05	0.001220128
NA		various ungrouped proteins	unclassified	-0.019633232	captive	25	25	1.62E-04	0.001810377
	15074	DNA integration	unclassified	-0.006792081	captive	25	25	2.06E-04	0.002237276
	3684	damaged DNA binding	unclassified	-0.002077954	captive	25	25	3.84E-04	0.003565724
	3697	single-stranded DNA binding	unclassified	-0.001769142	captive	25	25	4.56E-04	0.004147967
	6281	DNA repair	unclassified	-0.003367525	captive	25	25	4.73E-04	0.00416063
	6730	one-carbon metabolic process	unclassified	-0.001370097	captive	25	25	4.76E-04	0.00416063
	9055	electron transfer activity	unclassified	-0.001850054	captive	25	25	5.05E-04	0.004190296
	3824	catalytic activity	unclassified	-0.004211514	captive	25	25	5.20E-04	0.004215482
	51301	cell division	unclassified	-0.003044038	captive	25	25	5.33E-04	0.004242426
	16020	membrane	unclassified	-0.002691124	captive	25	25	7.12E-04	0.005381743
	9291	unidirectional conjugation	unclassified	-0.003178528	captive	25	25	7.91E-04	0.005880032
	6260	DNA replication	unclassified	-0.002750348	captive	25	25	0.001036897	0.007225879
	4553	hydrolase activity, hydrolyzing O-glycosyl compounds	unclassified	-0.001864653	captive	25	25	0.001125669	0.007606796
	16616	oxidoreductase activity, acting on the CH-OH group of donors, NAD or NADP as acceptor	unclassified	-0.00160965	captive	25	25	0.001197302	0.007970099
	16021	integral component of membrane	unclassified	-0.008961734	captive	25	25	0.001222809	0.008020192
	9252	peptidoglycan biosynthetic process	unclassified	-0.002688739	captive	25	25	0.001350523	0.008604758
	6261	DNA-dependent DNA replication	unclassified	-0.001608591	captive	25	25	0.001691071	0.010224798
	6520	cellular amino acid metabolic process	unclassified	-0.001403588	captive	25	25	0.001731714	0.010224798
	8360	regulation of cell shape	unclassified	-0.002754059	captive	25	25	0.001742342	0.010224798
	7049	cell cycle	unclassified	-0.002483261	captive	25	25	0.002245265	0.013005043
	31419	cobalamin binding	unclassified	-0.002670505	captive	25	25	0.00267261	0.015088404
	8168	methyltransferase activity	unclassified	-0.002584238	captive	25	25	0.003078589	0.016951245
	16887	ATPase activity	unclassified	-0.0028958	captive	25	25	0.003610392	0.01940042
	50662	coenzyme binding	unclassified	-0.001537984	captive	25	25	0.004485496	0.022733311
	30246	carbohydrate binding	unclassified	-0.001958177	captive	25	25	0.004754539	0.023662998
	16740	transferase activity	unclassified	-0.003118563	captive	25	25	0.005109702	0.025043154
	8094	DNA-dependent ATPase activity	unclassified	-8.25E-04	captive	25	25	0.00549128	0.026334526
	15991	ATP hydrolysis coupled proton transport	unclassified	0.001080266	wild	25	25	0.007792096	0.035103787
	16903	oxidoreductase activity, acting on the aldehyde or oxo group of donors	unclassified	-0.001090299	captive	25	25	0.007969202	0.035250673
	8137	NADH dehydrogenase (ubiquinone) activity	unclassified	-0.0015627	captive	25	25	0.007982776	0.035250673
	6351	transcription, DNA-templated	unclassified	-0.002693631	captive	25	25	0.009731135	0.039817305
	55085	transmembrane transport	unclassified	-0.002219435	captive	25	25	0.010508623	0.042607689
	5622	intracellular	unclassified	-0.002890151	captive	25	25	0.010773184	0.043286848

Table S3. Differentially abundant pathways between wild and captive rhino samples.

Pathway	Taxonomy	Coefficient	Greater_in	N	N not 0	P-value	Q-value
UNINTEGRATED	g Prevotella s Prevotella ruminicola	-0.051981171	captive	25	9	2.02E-05	9.92E-04
UNINTEGRATED	cumulative	-0.03305889	captive	25	25	0.001497569	0.022690913
UNINTEGRATED	g Ruminococcus s Ruminococcus flavefaciens	-0.030684375	captive	25	10	0.001604405	0.022690913
UNINTEGRATED	unclassified	-0.033446782	captive	25	25	0.001852319	0.022690913
UNMAPPED	cumulative	0.033255606	wild	25	25	0.002575304	0.024994181
UNINTEGRATED	g Ruminococcus s Ruminococcus albus	-0.027918194	captive	25	7	0.003060512	0.024994181
PWY_5103__L_isoleucine_biosynthesis_III	cumulative	-0.002217358	captive	25	25	0.007690747	0.046788991
PWY_6121__5_aminoimidazole_ribonucleotide_biosynthesis_I	cumulative	-0.001909641	captive	25	25	0.00800027	0.046788991
UNINTEGRATED	g Escherichia s Escherichia coli	0.012012033	wild	25	9	0.008593896	0.046788991
PWY_5103__L_isoleucine_biosynthesis_III	unclassified	-0.001874762	captive	25	25	0.021912986	0.100452787
PWY_5100__pyruvate_fermentation_to_acetate_and_lactate_II	unclassified	0.001541674	wild	25	25	0.022550626	0.100452787
PWY_2942__L_lysine_biosynthesis_III	cumulative	-0.001755957	captive	25	25	0.026088608	0.106528481
PWY_6122__5_aminoimidazole_ribonucleotide_biosynthesis_II	cumulative	-0.002021739	captive	25	25	0.03359139	0.117569866
PWY_6277__superpathway_of_5_aminoimidazole_ribonucleotide_biosynthesis	cumulative	-0.002021739	captive	25	25	0.03359139	0.117569866
PWY_7111__pyruvate_fermentation_to_isobutanol__engineered_	unclassified	-0.002506379	captive	25	25	0.042207739	0.130065889
PWY_1042__glycolysis_IV__plant_cytosol_	unclassified	-0.002183342	captive	25	25	0.043189036	0.130065889
PWY_6121__5_aminoimidazole_ribonucleotide_biosynthesis_I	unclassified	-0.001587175	captive	25	25	0.0451249	0.130065889
PWY_1042__glycolysis_IV__plant_cytosol_	cumulative	-0.001970994	captive	25	25	0.059376112	0.161634972
BRANCHED_CHAIN_AA_SYN_PWY__superpathway_of_branched_amino_acid_biosynthesis	cumulative	-0.001659963	captive	25	25	0.062958373	0.161941669
PWY_6122__5_aminoimidazole_ribonucleotide_biosynthesis_II	unclassified	-0.001871165	captive	25	25	0.069403573	0.161941669
PWY_6277__superpathway_of_5_aminoimidazole_ribonucleotide_biosynthesis	unclassified	-0.001871165	captive	25	25	0.069403573	0.161941669
PWY_5100__pyruvate_fermentation_to_acetate_and_lactate_II	cumulative	0.001121854	wild	25	25	0.100646326	0.203857319
ILEUSYN_PWY__L_isoleucine_biosynthesis_I__from_threonine_	cumulative	-0.001522005	captive	25	25	0.104790976	0.203857319
VALSYN_PWY__L_valine_biosynthesis	cumulative	-0.001522005	captive	25	25	0.104790976	0.203857319
VALSYN_PWY__L_valine_biosynthesis	unclassified	-0.001760418	captive	25	25	0.105774343	0.203857319
PWY_7111__pyruvate_fermentation_to_isobutanol__engineered_	cumulative	-0.001789228	captive	25	25	0.10816919	0.203857319
SER_GLYSYN_PWY__superpathway_of_L_serine_and_glycine_biosynthesis_I	cumulative	-0.001336398	captive	25	25	0.11703287	0.212392987
ILEUSYN_PWY__L_isoleucine_biosynthesis_I__from_threonine_	unclassified	-0.001645289	captive	25	25	0.127189722	0.216075835
BRANCHED_CHAIN_AA_SYN_PWY__superpathway_of_branched_amino_acid_biosynthesis	unclassified	-0.001551095	captive	25	25	0.127881616	0.216075835
PWY_6700__queuosine_biosynthesis	cumulative	-0.001428683	captive	25	25	0.168691268	0.275529071
PWY_6126__superpathway_of_adenosine_nucleotides_de_novo_biosynthesis_II	cumulative	0.001136724	wild	25	25	0.206033652	0.310279838
PWY_6126__superpathway_of_adenosine_nucleotides_de_novo_biosynthesis_II	unclassified	0.00107952	wild	25	25	0.20834578	0.310279838
DTDPRHAMSYN_PWY__dTDP_L_rhamnose_biosynthesis_I	cumulative	0.001336018	wild	25	25	0.217050274	0.310279838
UNINTEGRATED	g Bacteroides s Bacteroides fragilis	0.019161447	wild	25	5	0.224062929	0.310279838
UNINTEGRATED	g Shigella s Shigella sonnei	0.009169998	wild	25	3	0.225105226	0.310279838
DTDPRHAMSYN_PWY__dTDP_L_rhamnose_biosynthesis_I	unclassified	0.001405766	wild	25	25	0.227960697	0.310279838
UNINTEGRATED	g Streptococcus s Streptococcus suis	0.010185154	wild	25	5	0.252782523	0.334766044
PWY_7221__guanosine_ribonucleotides_de_novo_biosynthesis	cumulative	-0.001354599	captive	25	25	0.270408533	0.343548498
UNINTEGRATED	g Enterococcus s Enterococcus casseliflavus	0.007701956	wild	25	3	0.273436559	0.343548498
PWY_5104__L_isoleucine_biosynthesis_IV	cumulative	-8.05E-04	captive	25	25	0.307470258	0.376651066
PWY_6700__queuosine_biosynthesis	unclassified	-0.001155665	captive	25	25	0.355292419	0.41398152
PWY_7219__adenosine_ribonucleotides_de_novo_biosynthesis	cumulative	0.00125647	wild	25	25	0.358318576	0.41398152
UNINTEGRATED	g Streptococcus s Streptococcus gallolyticus	0.008800001	wild	25	5	0.363289905	0.41398152
PWY_7219__adenosine_ribonucleotides_de_novo_biosynthesis	unclassified	0.001338645	wild	25	25	0.405586489	0.451675863
SER_GLYSYN_PWY__superpathway_of_L_serine_and_glycine_biosynthesis_I	unclassified	-5.54E-04	captive	25	25	0.573042007	0.623979074
PWY_7229__superpathway_of_adenosine_nucleotides_de_novo_biosynthesis_I	unclassified	2.78E-04	wild	25	25	0.720228765	0.767200206
TRNA_CHARGING_PWY__tRNA_charging	cumulative	-2.48E-04	captive	25	25	0.781245813	0.814490315
PWY_7229__superpathway_of_adenosine_nucleotides_de_novo_biosynthesis_I	cumulative	-1.02E-04	captive	25	25	0.892750539	0.911349508
TRNA_CHARGING_PWY__tRNA_charging	unclassified	-7.56E-05	captive	25	25	0.941709492	0.941709492
